# The complete mitochondrial genome of Pacific golden plover *Pluvialis fulva* (Charadriiformes, charadriidae)

**DOI:** 10.1080/23802359.2016.1225524

**Published:** 2016-09-18

**Authors:** Jingjing Ding, Wei Liu, Yi Zhang, Qing Chang, Chaochao Hu

**Affiliations:** a Geographical Science Institute, NanJing Normal University, Nanjing, Jiangsu, China;; b Jiangsu Key Laboratory for Biodiversity and Biotechnology, College of Life Sciences, Nanjing Normal University, Nanjing, Jiangsu, China;; c Analytical and Testing Center, Nanjing Normal University, Nanjing, Jiangsu, China;; d Jiangsu Academy of Forestry, Nanjing, Jiangsu, China

**Keywords:** Mitogenome, *Pluvialis fulva*, charadriiformes

## Abstract

Pacific golden plover *Pluvialis fulva* (Charadriiformes, Scolopacidae) is obligate long-distance migrant bird breeding from northernmost Asia into western Alaska, and wintering on islands in the Pacific Ocean and Australia. In this study, we report the complete mitochondrial genome of *P. fulva*, which is a circular molecule of 16,854 bp in size and consists of 13 protein-coding genes, 2 ribosomal RNAs, 22 transfer RNAs, and a control region. The A + T content of the overall base of the composition of H-strand is 54.88% (A: 31.48%, T: 23.40%, C: 31.48%, and G: 13.65%). It is very interesting that there are some insertions/deletions in the *cytb* gene sequence (total of 10bp deletions and 5bp insertions in four positions), which may result in reading frame shift and/or internal stops. Some short microsatellite-like repeat regions (ACCACCC) are scatter in the control region. The phylogenetic analysis resolved a well-supported clade of Charadriiformes in which *Pluvialis* appears to be sister group of *Vanellus*. This mitogenome provides a valuable resource for further study of molecular systematics, species identification, population genetics, phylogeography, and conservation genetics of Charadriiformes.

Pacific golden plover *Pluvialis fulva* (Charadriiformes, Scolopacidae) is strongly migratory, with different populations travelling on narrow or broad front. Despite the fact that the population trend appears to be decreasing, it is evaluated as Least Concern (BirdLife International 2012). Many studies on morphology, ecology, behaviour, bioenergetics, and migration had been reported (Johnson et al. 2001, 2009; Withrow & Winker 2014; Jukema et al. 2015), but the basic genetics data are relatively unclear, and the taxonomic assignment has been debated recently (Baker et al. 2012; Withrow & Winker 2014). In this study, we sequenced the complete mitochondrial genome of *P. fulva*.

The complete mitogenome of *P. fulva* has been sequenced to better understand the mitogenomic characteristics and its phylogenetic relationships within Charadriiformes. The muscle specimen of *P. fulva* was collected from the costal of Rudong Country, Jiangsu Province, China (32°32′43.42″ N, 121°06′09.02″ E). A voucher specimen was preserved in absolute ethanol at Nanjing Normal University (NJNU: Pfu-2015007), Nanjing, China. Total DNA was extracted with standard phenol–chloroform methods according to Sambrook and Russell (1989).The complete mitochondrial genome was amplified and sequenced by 13 pairs of primers. Annotations were confirmed by comparing 17 Charadriiformes species with MITOS-generated annotations (Bernt et al. 2013).

The circular genome is 16,854 bp in length with 13 protein-coding genes, 2 ribosomal RNAs (12S rRNA and 16S rRNA), 22 transfer RNA genes, and a noncoding region. The overall nucleotide composition was A: 31.48%, T: 23.40%, C: 31.48%, and G: 13.65%. The annotated mitogenome of *P. fulva* is available online in NCBI (GenBank accession number: KX639757). It is very interesting that there are some insertions/deletions in the cytb gene sequence (total of 10bp deletions and 5bp insertions in four positions) which may result in reading frame shift and/or internal stops.

Seventeen closely related Charadriiformes mitogenomes (82.37%–86.43% identical bases) were downloaded and aligned with Clustal X 1.81 (Thompson et al. 1997; Strasburg, Alsace, France) using default parameters. There were 6572 (57.59%) conserved sites, 3612 (31.65%) parsim-info sites, and 1213 (10.63%) singleton sites. Total of 11,412 bp (with the concatenated 13 protein-coding genes) were used for phylogenetic analyses. The Bayesian phylogenetic analyses were performed using MrBayes 3.1.2 (Ronquist et al. 2012). Models of molecular evolution were assessed using MrModeltest 2.3 (Nylander 2004). The Four Markov Chains Monte Carlo (MCMC) chains were run for 1.0 × 10^6^ generations. Two independent runs were performed to allow additional confirmation of the convergence of MCMC runs.

The phylogenetic analysis (Figure 1) resolved a well-supported clade of Charadriiformes in which *Pluvialis* appears to be a sister group of *Vanellus*. The result indicated that there was great mitochondrial divergence within the Charadriiformes. The newly described mitogenome can be used in the future to disentangle the taxonomy within Plover, and provided a valuable resource for the studies related to shorebird’s molecular population genetics, phylogeography, and systematics.

**Figure 1.**
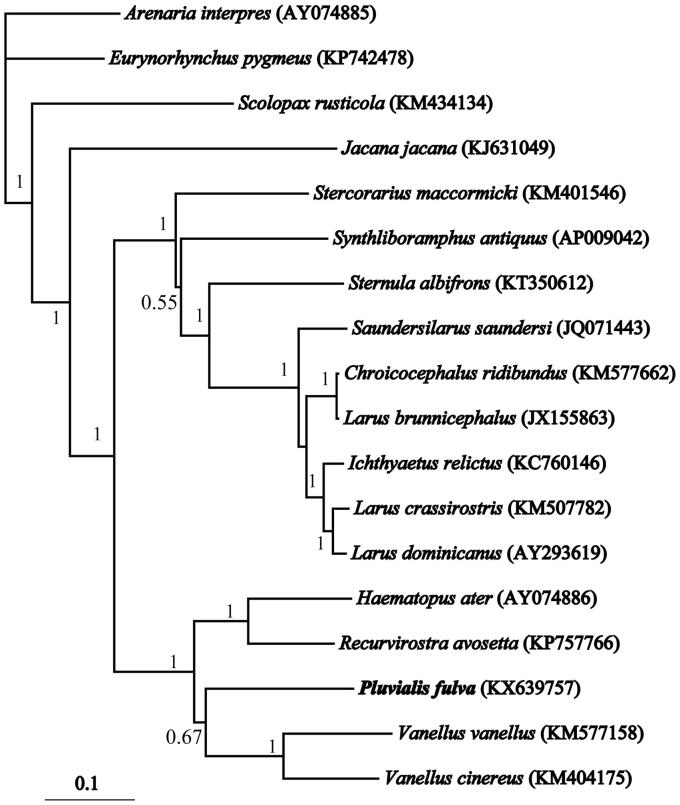
The unrooted tree of the Bayesian phylogenetic analysis of *P. fulva* and closely related 17 mitochondrial sequences. Numbers above each branches are the posterior probabilities. Genbank accession numbers of species are shown in parentheses.
